# Sinako, a study on HIV competent households in South Africa: a cluster-randomised controlled trial protocol

**DOI:** 10.1186/s13063-020-4082-0

**Published:** 2020-02-10

**Authors:** Caroline Masquillier, Lucia Knight, Linda Campbell, Neo Sematlane, Anton Delport, Tanyaradzwa Dube, Edwin Wouters

**Affiliations:** 1 Department of Social Sciences, University of Antwerp, Antwerp, Belgium; 2 School of Public Health, University of the Western Cape, Cape Town, South Africa

**Keywords:** People living with HIV, Household, HIV competency, Antiretroviral treatment, Community health workers, Cluster-randomised controlled trial, South Africa

## Abstract

**Background:**

With 7.7 million South Africans currently infected with human immunodeficiency virus (HIV) and 4.8 million currently receiving antiretroviral treatment (ART), the epidemic represents a considerable burden for the country’s resource-limited health system. In response to the health and human resource shortages, task shifting to community health workers (CHWs) and empowering people living with HIV (PLWH) are integral parts of a sustainable ART strategy. Despite the success of the ART programme, South Africa still faces both prevention and treatment challenges. To tackle these challenges, future endeavours need to focus on the role played by the households of PLWH in mediating between the community and PLWH themselves. Building health-enabling “HIV competent” households with the capacity to actively stimulate lifestyles that foster health, offers a potential strategy to tackle South Africa’s HIV-related challenges. The aim of the “*Sinako*: Households and HIV” study is to investigate to what extent and how an intervention can increase HIV competence in PLWH and their households, and subsequently optimise the impact of CHW support on individual ART outcomes.

**Methods:**

The “*Sinako*” study is a cluster-randomised controlled trial with two arms. In the control arm, CHWs offer a standard package of support to PLWH during home visits, focused on the individual. The intervention arm includes both a focus on the individual and the household to enable the patient to self-manage their treatment within an HIV competent household.

A longitudinal mixed methods design is adopted to analyse the data. For the quantitative data analysis, methods including latent cross-lagged modelling, multilevel modelling and logistic regression will be used. To assess the acceptability and feasibility of the intervention and to construct a comprehensive picture of the mechanisms underlying the impact on the household and the PLWH, qualitative data (in-depth interviews and focus group discussions) will be collected and analysed.

**Discussion:**

Stimulating HIV competence in households could be a feasible and sustainable strategy to optimise the outcomes of CHW interventions and thus be important for HIV treatment interventions in resource-limited settings.

**Trial registration:**

Pan African Clinical Trial Registry, PACTR201906476052236. Registered on 24 June 2019.

## Introduction

### Background

To date, 76.1 million people have become infected with human immunodeficiency virus (HIV) and the virus has claimed an estimated 35.0 million lives globally [1]. The HIV epidemic therefore remains one of the world’s largest public health challenges. In absolute numbers, South Africa bears the brunt of the epidemic, with about 7.7 million people living with HIV (PLWH) [2]. Considerable efforts have been made to address the epidemic; 4.8 million PLWH are currently receiving life-saving antiretroviral treatment (ART) in South Africa [3]. This number is further increasing with the adoption of the Universal Test and Treat (UTT) programme. UTT recommends treatment initiation for all people who are HIV positive, regardless of their CD4 count [2]. The massive ART scale-up has transformed HIV into a manageable, chronic illness, shifting HIV care from terminal care to chronic disease management [4].

Transition to a chronic care model is, however, placing an immense burden on the resource-limited health system. Recent estimates suggest that 42.7% of health professional posts in South Africa are vacant [5]. High attrition, underfunded public sector posts, and inefficient management and recruitment processes contribute to the shortage of health professionals [6]. As a response to these human resource shortages, innovative means of delivering long-term care for PLWH have been developed, such as “task shifting” [7]. Task shifting refers to “a process of delegation whereby tasks are moved, where appropriate, to less specialised health workers” [7]. There is clear evidence of the success of this delegation of HIV services from doctors to non-physician clinicians (type I); from clinicians to nurses (type II); from nurses to lay community health workers (CHWs) (type III); and eventually to self-management by PLWH themselves (type IV) [7].

Despite the success of the ART programme and task shifting, South Africa is still faced with challenges in terms of prevention as well as treatment. With respect to prevention, incidence rates remain high, with about 240,000 new HIV infections in 2018. Furthermore, 700,000 PLWH do not know their status and are therefore not enrolled into care or receiving treatment [1, 3]. Treatment challenges include low rates of adherence and retention in care; only 62% of PLWH are on ART [3], and 23% of patients enrolled in the South African ART programme disengaged from care at least once within a 2-year period [8]. In 2018, an estimated 71,000 PLWH died from acquired immune-deficiency syndrome (AIDS)-related causes [3]. These issues highlight the need to develop innovative and sustainable responses to successfully bring an end to the HIV epidemic in South Africa [1].

To provide effective preventive actions and chronic disease care within the climate of human resource shortages, it is not enough to simply shift responsibility for chronic HIV care to the community (type III) and PLWH (type IV) themselves. Future endeavours need to focus on the search for innovative ways to provide social support in response to these scarcities. A potential source of such support already exists at the intermediate level, between the community (type III) and PLWH (type IV), namely PLWH households. In this study, households are defined as a “co-residential unit, usually family-based in some way, which takes care of resource management and the primary needs of its members” [9].

The intermediate household level is often overlooked in the current chronic disease care delivery model. However, PLWH seldom live in isolation, and their home life is generally regarded as the closest and most basic context for individual development [10]. Previous research has shown that spatial proximity and day-to-day interactions are two characteristics that are vital to the daily provision of care and support to HIV-affected individuals [9]. The direct and indirect impact of transmission risk, care burden, social stigma, physical illness and emotional distress is shouldered by various household members [11]. Living with a chronic illness, such as HIV, is “complex and requires integration of self-management behaviours into the lifestyles of individuals and households” [12]. “Building health-enabling households” that support patients along the care continuum and stimulate positive living offers a potential strategy to tackle the current HIV treatment and prevention challenges [13].

### Theoretical framework

#### HIV competence and health-enabling contexts

In order to address prevention and treatment challenges within the household context extensive efforts are required to increase HIV knowledge, reduce stigma, stimulate HIV testing, improve health care-seeking behaviour, and encourage safe sexual practices—described by UNAIDS and other authors as the need for HIV competence [14, 15]. Achieving HIV competence cannot be done by individuals alone—it is a group phenomenon, since health decisions are seldom made in isolation [14]. HIV competence reflects the idea that “the likelihood that people will choose health-enhancing practices depends not only on individual-level factors, but also on the extent to which they live in social environments that enable and support this choice” [13].

These ideas are rooted in the ecological approach of Kelly et al. [16] with its emphasis on the importance of developing contexts that provide support in strengthening the individual and building social resources to respond to challenges [17]. Such a health-enabling context is assumed to be a responsive environment that stimulates individuals to become health competent by facilitating health-enhancing behaviours and promoting the spread of health information and health norms [16, 18]. In addition, a health-enabling context can support “collective efficacy”, meaning that people can take more control over their lives and their living environment in order to achieve better health [18]. This collective dimension is especially relevant for HIV as “HIV infects individuals and simultaneously affects a whole network of significant relationships” [19].

#### HIV competent households

Integrating the elements of this theoretical framework, the research team developed the theoretical concept of “HIV competent households” based on qualitative research [20] to lay the foundations for this project. In this conceptualisation of HIV competent households, it is shown that the household has the potential to form a health-enabling environment for PLWH. An HIV competent household is an environment in which the patient can be supported across the HIV care continuum in a sustainable manner. HIV competent households 1) gain, share and translate HIV-related knowledge into good prevention and treatment behaviour; 2) create a safe space for disclosure and dialogue about HIV; 3) foster ownership of HIV and responsibility for safe sexual practices, testing and ART; 4) build solidarity to support PLWH in its midst to adhere to ART and remain in care; and 5) are receptive to outside support (e.g. from a CHW) [20].

However, the road to HIV competence in the household is precarious and prone to obstacles at both the individual and household level. As a result of HIV-related stigma both outside and inside the household, the development of HIV competence can easily be undermined. Furthermore, a household’s lack of social support or emotional connectedness, discrimination against HIV or misconceptions about the illness can further inhibit the development of HIV competence at the household level and may even produce a health-impeding context [20]. In such a negative household environment, PLWH might not disclose their status, fearing stigmatisation. Non-disclosure may not only impact ART adherence, but also impacts practices for prevention of transmission to other household members [20]. This potentially health-impeding role of the household underlines the need for comprehensive and context-specific interventions to stimulate HIV competence at the household level [21].

#### The positive communication process as a mechanism of change

Based on the circumplex model of marital and family systems by Olson and the results of qualitative research by Masquillier et al., the research team developed the positive communication process (P^2^CP), which delineates four steps in the process of building HIV competence at the household level [22–24].

The road to HIV competency commences with the recognition of the reality of HIV by the PLWH themselves (P^2^CP step 1). A necessary condition for the building of HIV competence in the household is then disclosure, as household members can only offer social support related to living with HIV when the patient has shared their status (P^2^CP step 2) [25]. Equipped with the correct knowledge and communication skills provided by outside support (e.g. from a CHW), the patient and the confidant are encouraged to become change agents in the household (P^2^CP step 3). They create awareness and openness about the illness in their midst and the need for behaviour change to prevent further transmission to others. These change agents are therefore the motor that will prompt the move towards HIV competence at the household level. Moreover, the change agents will act as “household health advisors” by translating their knowledge and communication skills into positive HIV-related communication dynamics at the household level, such as safe sex negotiation or a conversation about adherence support. An increase in HIV-related knowledge supports the gradual process of normalisation of HIV in the household, which is required to build an environment that is responsive to HIV treatment and prevention. Finally, these constructive household dynamics are translated into HIV competence (P^2^CP step 4), resulting in a household that forms a health-enabling environment in which it is easier for the patient to self-manage their treatment, adhere to ART, and reduce the likelihood of a new HIV infection within the household (for instance, by increased condom use).

### Research aims

In this project, the HIV competent household concept will be advanced beyond the merely theoretical and conceptual level. Building on the existing literature and our preparatory work, the current project aims to investigate empirically to what extent and in what way HIV competent households can become sustainable health-enabling contexts that can provide an answer to the HIV prevention and treatment challenges facing South Africa. To this end, this project—the *Sinako* (‘we can’ in isiXhosa) HIV and households study—aims to test an evidence-based household intervention delivered by CHWs to: 1) increase HIV competence in PLWH and their households; and subsequently 2) optimise the impact of CHW support on individual antiretroviral treatment outcomes.

## Methods/design

### Trial design

The primary activity for the Sinako study will be the implementation and assessment of the impact of an HIV competent household intervention. Table 1 represents a schedule of intervention implementation, assessment and dissemination. The intervention will be assessed in a cluster-randomised control trial (RCT) with two arms. CHWs will be the primary actors in this RCT, either continuing to deliver a standard ART adherence support service (arm 1), or a standard ART adherence support service plus a household P^2^CP intervention (arm 2). CHWs, however, are linked to a health care facility, which creates a risk of contamination when CHWs active in different arms operate from the same facility. The facility was therefore selected as the cluster unit of randomisation. The health care facilities in the study setting were categorised as large or small facilities according to the numbers of CHWs employed, which also corresponds to the number of patients. Subsequently, 12 facilities were grouped by selected subdistricts and were randomly selected from the list of facilities for inclusion in the study in arms 1 and – resulting in six facilities or clusters per trial arm. Further blinding of the study arms was not possible because of the clear differences between the intervention and the standard of care. The design and report of this clinical trial protocol follows the Standard Protocol Items: Recommendations for Interventional Trials (SPIRIT) statement (Additional file 1).

**Table 1:** Schedule of intervention implementation, assessment and dissemination

	Year 1	Year 2	Year 3	Year 4
Preparatory research activities				
Ethical approval	X			
Preparatory literature review	X			
Quantitative survey development	X			
Mobile data collection tool programming	X			
Qualitative questionnaire development		X		
Development of the household intervention	X	X		
Pilot of the household intervention		X		
Drawing study sample for cluster-RCT		X		
Fieldworker training		X		
Baseline assessment of HIV competence and HIV outcomes				
Baseline quantitative data gathering, capturing and cleaning		X		
Qualitative data collection		X	X	
Baseline quantitative data analysis			X	X
Writing of reports and articles		X	X	X
Implementing the household intervention				
Workshops providing training to CHWs conducting the intervention		X		
Screen participants for eligibility		X		
Informed consent		X		
Implementing the intervention in the experimental households		X	X	
Monitoring of the intervention using qualitative methods		X	X	
Measuring the impact of the household intervention				
Developing a quantitative postintervention survey		X		
Follow-up quantitative data gathering, capturing and cleaning		X	X	
Focus group discussions on experience of intervention implementation			X	
Follow-up quantitative data analysis			X	X
Qualitative data analysis			X	X
Writing of report and articles			X	X
Dissemination of knowledge on the household competence intervention				
Presenting results at international conferences		X	X	X
Writing of report and articles		X	X	X
Finalising PhDs				X

*CHW* community health worker, *HIV* human immunodeficiency virus, *PhD* Doctor of Philosophy, *RCT* randomised controlled trial

### Study setting and site selection

The cluster-RCT will be executed in the Western Cape Province of South Africa in five health subdistricts of the Cape Metro area: Khayelitsha, Klipfontein, Mitchell’s Plain, Eastern and Western.

Mitchell’s Plain has the largest population of the five subdistricts and is predominantly inhabitated by of Coloureds (91%). In contrast, the population in Khayelitsha is mainly Black African (99%). For the three other subdistricts, race distribution is almost equal between Coloureds and Black African populations, with the Western subdistrict showing a significant presence of the White population (29%). All these subdistricts are confronted with severe social and economic challenges, and poverty is widespread. Unemployment is omnipresent with an average unemployment rate of 28.6%, ranging from 18% in Western to 38% in Khayelitsha. Of the five subdistricts, Khayelitsha is the most impoverished, with more than half of the households living in informal dwellings (55%) and with 74% of households surviving on a monthly income of R3200 or less (approximately US$218). Conversely, the Western subdistrict appears to be a little less disadvantaged than the other five subdistricts, with 15% of households living in informal dwellings and a slightly lower unemployment rate of 18% [26].

These social and economic challenges translate into health-related challenges as a result of limited access to health care, education, intra-partner violence, and transactional sex, among others [27]. According to the national antenatal sentinel HIV and syphilis survey, HIV prevalence in the Cape Town metropolitan area, within which the five subdistricts are located, was 21.6% [28]. Moreover, across the entire Western Cape Province, Khayelitsha was identified as having the highest HIV prevalence, with a seroprevalence of 34.3% among pregnant women [29]. With regard to retention in care after 12 months for ART, Eastern performs best of all five subdistricts included in the study at 71.7%, while Klipfontein has demonstrated the least favourable percentage of retention of patients in care, with 61.6% of the patients retained after 12 months. At 48 months of retention in ART care, a similar pattern can be found: Eastern (58.9%); Mitchell’s Plain (58.5%); Klipfontein (56.3%); Western (56.3%); and Khayelitsha (55.0%) [30].

#### Participants

Inclusion criteria for participants include the following: a minimum age of 18 years; having commenced ART within 4 weeks of enrolment either for the first time or again in the case of previous defaulting; having a household member above 18 years old; not being co-infected with tuberculosis at the time of the test; not tested as a result of pregnancy; accessing HIV care and treatment at one of the designated health care facilities for this cluster-RCT; and living in the area of this facility.

The CHWs participating in this trial were selected through an application process based on various criteria, including: having experience as a CHW supporting people living with HIV; willingness to learn new skills and embrace different methods for supporting ART adherence; and willingness to work in the community, including locating clients and conducting the intervention in the client’s household. Intensive training workshops have been held to train the CHWs recruited in the intervention arm of the RCT about their specific tasks and equip them with the skills to deliver the intervention.

#### Sampling

A sample size of 180 individuals per arm was obtained by sampling 12 clusters in total (six for each arm) with 90% power to determine an increase in ART adherence (primary outcome (see below)) from 68% to 83% (effect size = 15%) postintervention over a period of 12 months. The proportion in arm 1 (the control group) is assumed to be 0.68 under the null hypothesis and 0.83 under the alternative hypothesis. This sample size has been calculated for a two-sided Z test (unpooled) and 5% significance level. The intracluster correlation is 0.0020. The estimated effect size is conservative since this is a new intervention that has not been rigorously assessed in South Africa. We hope to increase the ART adherence levels by 30%; however, we have powered the study on a lower effect size (15%) to avoid a type 2 error. The total sample size of 640 individuals (320 per arm) instead of 360 (180 per arm) will be used to allow for loss to follow-up. As a result of this oversampling to account for loss to follow-up during the trial, CHW will deliver the intervention to a total of 320 PLWH receiving ART adherence support.

#### Recruitment

As part of the standard procedure in the South African health system, the counsellor tests the individual at the clinic. Upon a positive HIV test, the counsellor opens a patient file and scans the patient’s preliminary eligibility criteria. A second clinic visit is then scheduled where the patient will be introduced to their CHW and the study. If the patient refuses to participate, the patient stays in the regular South African health system. If the potential participant agrees, the CHW enrols the individual and schedules a first visit at home or an alternative location preferable to them. At this home visit, the CHW provides further information about the study, invites the PLWH to complete an informed consent form, reassures confidentiality and explains the importance of an interview with a household member. The CHW takes household member names and then schedules the second home visit where the individual baseline interview will be conducted by the fieldworker. The household includes all those people who “eat from the same pot” for at least four nights per week over the past month. Based on the household information document, household members will be randomised by the fieldwork team for the household member interview. If the household member interview is not completed within 30 days of patient enrolment, the household member is not included in the study.

### Intervention

Before the start of the intervention visits, the PLWH in both the intervention and control arms receive similar preparatory visits.

#### Preparatory visits (month 1)

In the first preparatory visit, the study is introduced by the CHW and consent is asked to collect information about the PLWH and the contact details of the household members. Furthermore, in this first visit an assessment is made of the PLWH’s ART adherence (by doing a pill count) and household context (by doing a home assessment).

Between the first and second preparatory visit, a household member will be randomly selected from the information sheet collected by the CHW at the first visit and be contacted for a household interview. If the research team was not able to contact or interview the first randomly selected household member, then a second or third household member will be contacted. The interview with the household member must occur before the first intervention visit. If the research team fails to interview a household member within this time frame, no household member interview will be conducted related to that patient.

In a second preparatory visit, which is a standard of care visit that all patients enrolled in the study receive, an evaluation of the ART adherence (pill count) will be repeated. A fieldworker will accompany the CHW during this visit, and conduct and conduct afterwards a baseline interviews.

#### Intervention visits (months 2–8)

After completion of these initial preparatory visits, the intervention will start. In the control arm, the CHW will offer a standard package of support (i.e. a pill count) focused at the individual level. In the intervention arm, in addition to the pill count the CHW activities entail two additional components: 1) an additional individual-level component to stimulate the self-management skills of the HIV patient; and 2) a context-focused component for promoting HIV competent households.

**Fig. 1:**
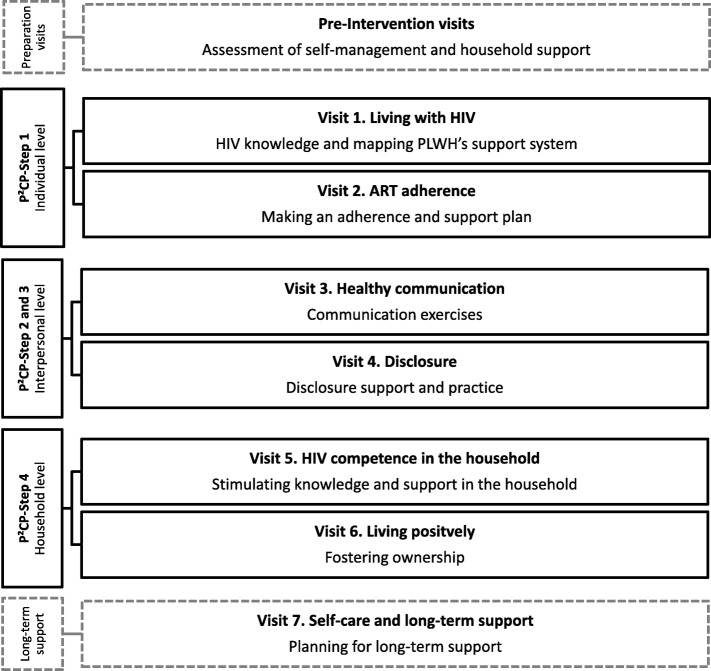
Intervention visits related to the Positive Communication Process (P²CP). ART antiretroviral treatment, HIV human immunodeficiency virus, P2CP positive communication process, PLWH person living with HIV

Figure 1 outlines an overview of the intervention visits. Intervention visits are instructive, sessional and deliberately structured to systematically deliver the intended content of the intervention. In between these main visits, time has been provided for catch-up visits in case a CHW could not finish a previous main visit. In the first two main visits (hereafter called visits) focus is placed on the individual level development. The third and fourth visits focus at the interpersonal level by examining healthy communication and disclosure. The fifth and sixth visits are household-level focused, aiming to stimulate HIV competency in the household. The seventh session completes this intervention by revisiting the previous sessions and developing a long-term household support plan.

Because disclosure is a critical component within HIV competent households and a common thread throughout delivery of the intervention, at the end of each and every intervention visit the PLWH will be encouraged to invite and, where possible, bring a household member or sexual partner to the next visit. If a new household member or sexual partner joins a session, their knowledge about HIV and ART will be assessed and improved by means of a visual HIV fact sheet. The household member/sexual partner will be included in the activities for the remainder of the intervention visit and be invited to join the next visits as well. If the PLWH did not bring a sexual partner or household member to the visit, the CHW will invite the PLWH to do so at the next visit. However, while highly encouraged to bring a household member, this is not a prerequisite to continue the intervention.

#### Piloting the HIV competent household intervention

The intervention pilot was undertaken with a small subsample of two ART patients in the intervention community from a facility not included in the RCT. The outcomes were incorporated into the final intervention. The feedback from the pilot included the need to allow for the possibility for “in between visits” to catch up on any activities that were not completed during the previous session. The pilot also demonstrated the need for more time than had been planned for CHW training. More engagement with the CHWs was therefore included in the training, with more role playing and simulated intervention time rather than time allocated for them to read the manual and self-learn.

#### Implementation of the HIV competent household intervention

The CHWs who are part of the intervention arm received 9 training days with a focus on role play and ensuring that the CHWs understand the intervention and the importance of standardisation of the delivery. At the end of the training, the CHWs were informally assessed to evaluate the grasp of content and technique to deliver the intervention. Furthermore, regular debriefing sessions are planned with the CHWs to monitor compliance with the intended standard of the intervention delivery and to improve adherence to the intervention protocols.

Various strategies are followed to maximise participant retention in the study. First, potential participants who return for follow-up care and treatment visits after the initial diagnosis visit are recruited into the study. Patients who return for follow-up treatment and care portray commitment to their own health betterment and are as such perceived as patients who are likely to commit to long-term participation in the Sinako study. Second, CHWs have been trained to answer pertinent questions about the study that may be posed by potential participants at every opportunity or as and when requested. Third, tailored strategies are used specific to the individual participant. For instance, a potential participant of the Sinako study will, from time to time, travel out of the province. This poses a risk of attrition from the study. To mitigate this risk and improve retention rates, the CHWs have been trained to check with the enrolled patients regarding travelling plans. In addition, the patient is also requested to inform the CHW of such travel, whether planned or occurring as an emergency. Finally, the CHW follow-up visits with the patient or the fieldworker data collection appointment with the household member are, as far as possible, scheduled according to the participants’ convenience. This is to ensure that study activities adapt to participants’ availability and are not imposed. This in turn is intended to improve retention.

If an individual participant does not want to continue participating in the study the intervention for this particular respondent will be stopped. In case of a particular adverse event (e.g. admission to a hospital), the principal investigator will make an informed decision whether or not to continue the trial for this particular participant.

### Participant timeline

**Fig. 2:**
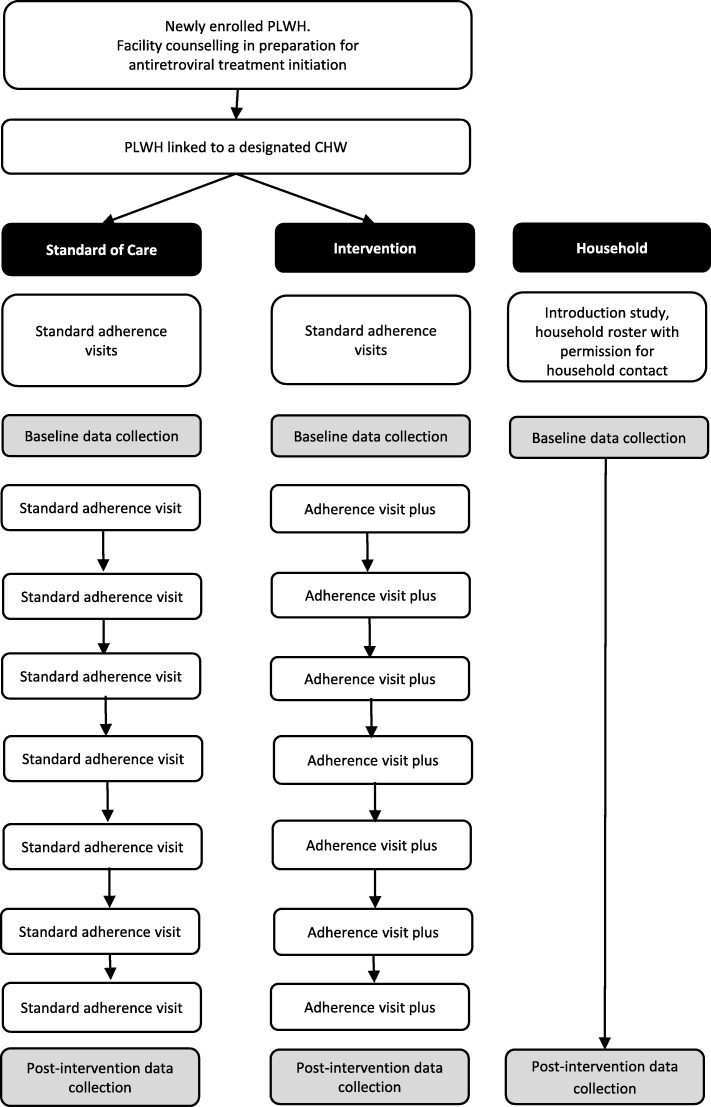
Participant timeline

Figure 2 shows details of the schedule of enrolment, intervention and assessment.

### Outcomes

In order to assess the impact of the intervention, key baseline assessment indicators are considered. These include primary and secondary outcomes (Table 2). As the current study aims to optimise the impact of CHW support on individual ART outcomes by stimulating HIV competent households, the primary outcomes for this study are: 1) patient’s viral load and 2) ART adherence (measured by the Adherence to Combination Therapy Questionnaire [31]). Data on ART adherence is collected from the enrolled individual by the research team. In addition, departmental permission has been requested to access the viral load data already collected by the Department of Health. As such, this study only requires secondary access; viral loads will not be collected by the research team.

**Table 2:** Outcomes for pre- and postintervention assessment

Primary outcomes	
• Viral load	
• Adherence to antiretroviral treatment	
Secondary outcomes	
Individual-level:	
• HIV knowledge	
• Condom use	
• Quality of life	
• Self-management	
• Disclosure	
• Perceived social support	
Household/-level:	
• HIV knowledge	
• Condom use	
• HIV-related stigma	
• Communication about HIV	
• Household functioning	
• HIV testing	
• Support to a household member living with HIV	

*HIV* human immunodeficiency virus

Moreover, we assess the impact of our intervention on a range of secondary process-related outcomes, measuring both individual outcomes and aspects of HIV competence of the households, which should facilitate individual ART adherence (and thus support the primary outcomes). At the individual level, the PLWH questionnaire assesses HIV knowledge, condom use, quality of life, self-management, perceived social support and disclosure. Outcomes measured at the household level include HIV knowledge, condom use, HIV-related stigma, communication about HIV, household functioning, HIV testing and support to a household member living with HIV. These outcomes are included as indications of household comfort with HIV and communicating about HIV and could be considered as proxy measures for HIV competency at the household level.

### Data collection

Methodologically, this study aims to analyse unique longitudinal data in a partially mixed concurrent equal status design, which involves “conducting a study that has two phases that occur concurrently, so that the quantitative and qualitative phases have approximately equal weight” [32]. Quantitative and qualitative longitudinal methods are both used in order to capitalise on the strengths of each approach. This methodological triangulation increases validity and produces a more comprehensive picture of the studied phenomena. Longitudinal qualitative research can provide explanation for patterns observed in quantitative datasets [33]. The quantitative and qualitative data collection occur simultaneously, but are analysed separately. The findings of both research methods are integrated in the theory formulation phase [32].

#### Quantitative data collection

The quantitative data collection is performed by experienced fieldworkers who have received additional training to sensitise them to the specifics of the study in order to ensure quality and standardisation of the research process. An innovative feature of this cluster-RCT is that one household member of each PLWH enrolled in the study is also interviewed. This enables collection of data on the level of HIV competence within the household (e.g. household functioning, communication, ownership, solidarity, acceptance of outside support). In order to avoid inadvertent disclosure, and to protect patient privacy, the interview with the household member is held at a different time and will be presented as a general health survey. Furthermore, patient and household interviews will be conducted by different interviewers who have experience with this research method in similar research contexts. Small tokens of appreciation in the form of a shopping voucher (for R75 (in the control arm) and R150 (in the intervention arm)) are provided to participants who complete the baseline and follow-up data collection.

##### Baseline data collection

For the baseline data collection, the CHW presents the study during the first pre-intervention visit and asks for informed written consent for obtaining contact information of the household members used for the household interview (see section on “Intervention” above). A trained fieldworker joins the CHW during the second preintervention visit. After obtaining informed consent from a PLWH willing to participate in the full study, the fieldworker administers the questionnaire on a hand-held device, using Mobenzi software [34].

##### Follow-up data collection

In order to ensure maximum comparability, the postintervention questionnaire collects information on the same outcomes as the baseline questionnaire from the same respondents in both the intervention and control arms of the RCT. The same data gathering procedure will be followed for the post-intervention assessment as for the baseline data collection. If a patient drops out, the research team will try to trace the patient to understand the reason for this loss to follow-up.

#### Qualitative data collection

In order to both produce a comprehensive picture of the mechanisms underlying the impact of the intervention on the household and PLWH, and to assess the feasibility and acceptability of the household intervention, different qualitative data sources are employed, namely focus group discussions with the CHWs and repeated in-depth interviews with PLWH in the intervention arm and interviews with household members who voluntarily took part in the intervention visits. These various qualitative research methods allow examination of the same topic from different angles, achieving data triangulation and improving validity of results [35].

In-depth interviews are used to reveal the internal dynamics of the household and the acceptability of the household intervention. A sample is drawn of approximately 15 PLWH in the intervention arm. This number is indicative, as qualitative data collection will continue until saturation has been reached. In addition, for each PLWH who participates, one household member (who voluntarily participated in the intervention) is invited for an interview. Using a semistructured interview guide, longitudinal in-depth interviews with the selected respondents will be conducted at three different time points: before the start of the intervention (month 0); in the middle of the intervention (month 6); and at the end of the intervention (month 12). Longitudinal qualitative data collection allows assessment of the changing dynamics within and outside the household that influence HIV competency, as well as its impact on PLWH and their (un)infected household members. The topics explored during the qualitative interview include HIV testing, stigma, disclosure, treatment adherence support, household support, and aspects of HIV competence. All interviews are conducted in the native language of the respondents (isiXhosa, Afrikaans or English).

Furthermore, all CHWs delivering the intervention are invited to participate in a focus group discussion to assess the feasibility of the intervention. The perceptions of those delivering the intervention are valuable because they may have important divergent insights into the way in which the intervention works to change HIV competence levels. The focus group discussions are conducted with all CHWs in their preferred language (isiXhosa, Afrikaans or English).

### Data processing and management

All quantitative and qualitative data will be anonymised and stored on a secure server. The list with the names of the respondents and their corresponding respondent numbers will be stored safely in a locked cabinet in the office of a School of Public Health or University of Antwerp researcher. The participant list will only be used for the purpose of identifying the follow-up respondents. This list with respondent names will be kept separate from the quantitative and qualitative databases. All these data will be kept for 5 years after the completion of the study.

#### Quantitative data processing and management

The quantitative data collection is guided by the mobile application Mobenzi. The Mobenzi servers are hosted in private subnets of the Amazon Web Service, where security group filters and network access control lists are utilised within a virtual private network (VPN) environment to ensure data security. Completed quantitative surveys are periodically uploaded and removed from the fieldworkers’ devices once the server acknowledges its receipt. Data are encrypted in transit using Secure Sockets Layer (SSL) [34].

#### Qualitative data processing and management

The qualitative in-depth interviews with PLWH and the focus group discussions with the CHWs delivering the intervention will be recorded. These data will be captured and analysed so that the anonymity of the respondents is maintained. Each respondent will be given a unique identifier. The coding sheet with all respondent numbers (identifiers) will be stored on a secure campus server. The audience will thus not be able to link individual statements to particular focus group participants and interviewees. If any statements would potentially reveal the identity of a respondent (e.g. because the respondent gives information specific to a certain household or patient), the research team will not include this statement so as to protect their identity.

### Data analyses

#### Quantitative data analysis

The comparison between the two arms using cluster-specific analysis techniques will allow us to assess the net impact of the household intervention on both the primary and secondary outcomes. First, we will perform an intent-to-treat analysis. In a second step, an analysis based on dose-response data will be conducted.

Furthermore, the main relationships between the relevant concepts (household intervention, HIV competence, prevention and treatment outcomes) will be analysed using latent cross-lagged modelling (Mplus). Using chi-square difference testing [36], measurement invariance will be tested to assess whether the latent factors are fully scalar invariant over time (pre- and postintervention). The latent factors will be modelled accordingly in the structural models over time [37]. The analyses will control for demographic and social characteristics, and the medical and medication history of the patient. Furthermore, other quantitative data analysis methods will be used to analyse the baseline and follow-up quantitative data, such as latent cross-lagged modelling, multilevel modelling, logistic regression and Poisson regression, among others. In this way, these results will allow us to successfully reach our research aims, namely to develop and scientifically test the impact of a household intervention on ART outcomes. No missing data are to be expected because of the nature of the Mobenzi data collection tool. To monitor the attrition between the first and second wave, an attrition analysis will be conducted.

#### Qualitative data analysis

Data collection and data analysis phases will be alternated to assist subsequent interviews and to assess when data saturation has been reached. After written informed consent is obtained all interviews will be audiotaped, allowing us to produce a detailed transcript of the interviews. These transcripts ensure accuracy of what is said and serve as the basis for data analysis. The recordings of the interviews will be transcribed verbatim and, when necessary, translated into English. A sample number of translations will be back translated into the local language for a quality check. Transcripts will be imported into NVivo. Data will be analysed carefully by reading and re-reading the field notes and transcripts of interviews. Codes for a sample of transcripts will be compared with another researcher’s codes and similarities and differences will be discussed, thus ensuring intercoder reliability. The analysis will be performed in accordance with the Grounded Theory principles described by Strauss and Corbin [38].

### Monitoring

When baseline data become available, descriptive analysis and structural equation modelling using Mplus will be conducted. A special data monitoring committee, made up of delegates from both institutions and external institutes, will be informed on the progress of the trial. This committee is independent from the sponsor and funders. Furthermore, the study will be guided by a steering committee, consisting of the two local principal investigators and a postdoctoral fellow.

A debriefing and internal monitoring plan will be followed to further monitor the intervention progress and to assess quality of delivery. Adverse events resulting from the intervention are reported on the same day to the principal investigator who will report these to the ethics committee of both institutions involved in the study (the University of the Western Cape and the University of Antwerp). However, no extreme adverse events are anticipated. In case psychological support is needed, the research team will provide counselling contacts.

One key to monitoring and evaluation is establishing whether it is ethical to continue the trial. To limit the potential risks for participants, we will organise a mid-term review of the intervention to assess its initial impact. In the unlikely event that the intervention has a negative impact on the health or mental well-being of the participants the trial will be stopped immediately. The project partners (the University of the Western Cape and the University of Antwerp) must mutually decide this in consultation with one another and the ethics committees of both institutions.

## Ethics, consent and permissions

Before study enrolment informed written consent of all participants is obtained. The consent forms are available in both English, isiXhosa and Afrikaans. The purpose of the study and its design and aspects such as informed consent and confidentiality is explained in an understandable manner to the respondent in the language of their preference (English, isiXhosa or Afrikaans). This information is also distributed by means of an information leaflet which the participants receive from the fieldworker. Written informed consent is required not only for study enrolment, but also for audio recording and for the publication of the findings. After written informed consent, respondents who agree to be included in the study are subjected to either a baseline and follow-up survey or a baseline and follow-up interview plus a household intervention.

Respondents can withdraw from the study at any time without penalty or loss of benefits to which they are entitled. If the respondent faces issues they do not want to discuss, the researcher will be sensitive to the interests of the participants by not pressing the issue and moving on to the next question.

### Ancillary and post-trial care

There are essentially three groups of participants: 1) HIV patients on ART; 2) household members; and 3) CHWs providing the intervention. Groups 2 and 3 are not exposed to any risks. These participants will share their views and experiences regarding life in the household (group 2) and their work (group 3), respectively.

The PLWH enrolled in the cluster-RCT (group 1) are exposed to two potential risks for which we have developed strategies to prevent and mitigate possible negative effects. First, the patients on ART receiving the standard treatment (CHW support) are not exposed to any potential negative effect. The Non-Governmental Organisation (NGO) providing the CHW support has been providing this support for several years and is accredited and funded by the provincial Department of Health. These trained CHWs have a standard procedure to protect the person living with HIV from any unintended consequences (e.g. the disclosure of their HIV status to the family/community). Patients starting treatment follow counselling sessions at the clinic where they are introduced to their CHW, who then makes an appointment for follow-up support visits (starting with a home assessment to record the social and housing conditions). If the patient does not want the CHW to visit their home, the meetings with the CHW are organised at their preferred location. This standardised procedure has been working for many years and has assisted thousands of HIV patients to commence their treatment. No unintended consequences are therefore expected in this arm. The HIV patients in the intervention arm, however, will be subjected to a household intervention. The intervention is based on the available literature on intervention development and the theoretical frameworks developed in family sociology and psychology [39]. The entire development process also incorporated all relevant stakeholders, namely HIV patients, CHWs, the Department of Health and the City of Cape Town. However, it must be noted that every intervention can have unintended (negative) consequences. When the CHW or fieldworkers deem necessary, they will also provide contact details for relevant referrals to health or social development government services or community-based or NGOs experienced in mitigating negative family dynamics and HIV treatment difficulties.

Second, it is possible that some of the participating HIV patients have not yet disclosed their HIV-positive status to their household members. For this reason, the patient interviews and the household interviews will be separated entirely. Both interviews will be executed on different dates and by different fieldworkers. The household interview will be framed as a general health survey in order to protect the privacy of the participating HIV patients.

After the completion of the study, the participants will be referred back into the health system. All patients revert to the standard of care delivered by the Department of Health, including facility visits and ART adherence clubs for stable patients.

## Dissemination of results and findings

The study results will be presented to the scientific community via journal publications and presentations at international conferences. People who are formally named and linked to the study and others who are directly involved, who have actively participated in the preparation or writing of the articles, are eligible for authorship. There is no intention to make use of professional writers. Furthermore, all relevant stakeholders will be informed of the research results; these include the Western Cape Department of Health, the City of Cape Town, the participating NGO and their CHWs, and patient representatives. The goal is to share the resulting knowledge with the relevant people who can subsequently adopt the (hopefully) successful interventions to improve CHW support for HIV patients on ART.

## Discussion

Despite the success of the ART programme, South Africa still faces both prevention and treatment challenges. To tackle these challenges, stimulating HIV competence at the household level could potentially be a feasible and sustainable strategy to optimise the outcomes of CHW interventions in a resource-constrained context. This paper provides an overview of the Sinako study. The aim of this cluster-RCT in South Africa is to investigate to what extent and how an intervention can: 1) increase HIV competence in PLWH and their households; and subsequently 2) optimise the impact of CHW support on individual ART outcomes. A longitudinal mixed methods design is adopted to analyse the data of the cluster-RCT Sinako study with two arms: 1) a control arm where CHWs will offer a standard package of support to PLWH during home visits which is only focused on the individual; and 2) an intervention arm where, during home visits, CHWs will focus on both the individual and the household in order enable the patient to self-manage their HIV treatment within an HIV competent household.

The Sinako study has to date encountered a couple of unexpected delays, stemming from policy changes in the field. In early 2019, the local Department of Health announced an amendment in operational arrangements with regards to NGOs and CHWs. These changes mainly relate to remuneration of CHWs. Originally, CHWs had been mainly employed on a 50% full-time equivalent basis by NGOs and remunerated from non-South African government funding and external sources of aid funding. The change in operation implied that the CHWs were now effectively employed by the government and remunerated from government funds, channelled through the NGOs, although administratively NGOs still provide oversight of CHWs. Prior to this change, the CHWs worked part-time for the Department of Health, and would therefore be able to take on part-time responsibilities for the study. However, the policy change resulted in the recruitment of full-time CHWs for the length of the intervention to work exclusively for the research project. This new strategy required a new recruitment process, which resulted in delays in the roll-out of the RCT.

## Trial status

The ethics committee of the University of the Western Cape (June 2019) and the ethical committee for the Social Sciences and Humanities of the University of Antwerp (September 2018) provided ethical approval for this study. Permission by the City of Cape Town was received in July 2019 and by the Western Cape Department of Health was granted by September 2019 for all but one facility, which was granted in December 2019. In this facility, the data collection started when approval was received.

## Protocol version

An application for funding was submitted in April 2017 to the Research Foundation Flanders and in May 2017 to the VLIR-UOS Research Foundation—Flanders for different aspects of this cluster-RCT. It went through thorough external peer review for each funding organisation separately. Funding was granted for 4 years, starting from January 2018. In November 2018, we applied for additional funding for the qualitative research component via a Global Minds scholarship at the University of Antwerp and in August 2018 for NRF funding. Each funding body reviewed various aspects of the qualitative research component separately. This article is based on the final protocol (version 1, June 2019). Recruitment for the baseline survey and intervention began in year 2 (8 October 2019). We anticipate that recruitment will be completed by year 3 (May 2020). The postintervention survey and the longitudinal qualitative work are expected to be finalised by year 3 (October 2020).

## Supplementary information

**Additional file 1.** Standard Protocol Items: Recommendations for Interventional Trials (SPIRIT) checklist.

**Additional file 2.** Consent forms.

## Data Availability

The access to the dataset is restricted at this point in time to the project research team members of the two research institutions (the University of the Western Cape and the University of Antwerp). The same applies to the statistical code. In due course, these may be made available by the University of the Western Cape principal investigator (LK), or the University of Antwerp principal investigator (EW) on reasonable request, and once clearance is obtained from the University of the Western Cape and the University of Antwerp.

## Abbreviations

AIDS: Acquired immune-deficiency syndrome
ART: Antiretroviral treatment
CHW: Community health worker
HIV: Human immunodeficiency virus
NGO: Non-Governmental Organisation
P^2^CP: Positive communication process
PLWH: Person living with human immunodeficiency virus
RCT: Randomised controlled trial
SPIRIT: Standard Protocol Items: Recommendations for Interventional Trials
UTT: Universal Test and Treat
