# Supplementary material for: Sinako, a study on HIV competent households in South Africa: a cluster-randomised controlled trial protocol
Source: Trials. 2020 Feb 10;21:154. doi: 10.1186/s13063-020-4082-0 (PMC7011384; doi:10.1186/s13063-020-4082-0)
Supplement: Supplementary file 2 — Additional file 2. Consent forms. [file 13063_2020_4082_MOESM2_ESM.zip › BASELINE INTERVENTION INT.docxXHOSA.pdf]

FACULTY OF COMMUNITY AND HEALTH SCIENCES

School of Public Health

Private Bag X17, Bellville, 7535
South Africa
Tel: +27 (0) 21 959 2809/2132
Fax: +27 (0) 21 9592872
Website:
http://www.uwc.ac.za/faculties/chs/soph

Universiteit Antwerpen

IPHETSHANA LENKCUKACHA: IINKCUKHANCA ZOLAWULO LOKUXHUMANA

Isihloko seProjekthi**: Amakhaya kwinkathelelo yeHIV: ukungenelela ukuze kuxhaswe indima ephakathi kwikhaya ekuncedeni uluntu ngononophelo lwe HIV.**

Mnumzana/Nenekazi elihloniphekileyo,

Lungantoni oluphando?

Thina abaphandi besikolo semfundo ephakamileyo sase Ntshona Koloni, kunye nabaphandi besikolo semfudo ephakamileyo i Antwerp(Belgium) senza uphando ngenkxaso yasemakhaya kubantu abaphila nentsholongwane ye HIV. Siyakumema uthabathe inxhaxheba koluphando kuba uvavanyo lwaa bonisa ukuba uphila nentsholongwane ka gawulayo kwaye uqalise unyango. Injongo yoluphando kukuqonda ngamava akho okuphila nentsholongwane kagawulayo kwakunye nokuba kunyango lwayo.

Ndiyakucelwa ukuba ndenze ntoni ukuba ndiyavuma ukuthatha inxaxheba?

Siyakumema ukuba uthabathe inxaxheba koluphando kuba igalelo lakho liyakusinceda ukuba siqonde indlela yokuxhasa abantu abaphila nentsholongwane ka gawulayo, nokuthi mhlawumbi kwixa elizayo incede abanye abantu abazibona bekwimeko ebunje ngale. Okokuqala singathanda ukwenza udliwano ndlebe nawe apho siyakukucela uphendule imibuzo ethile emalunga nawe, ikhaya lakho, uvavanyo lwentsholongwane ka gawulayo, ukuveza isimo sakho kwintsapho yakho, inkxaso yosapho lwakho kunye noluntu, nezinye izinto ezibalulekileyo malunga nokuphila nentsholongwane kagawulayo.

Ekupheleni koluphando singathanda ukuba ube yinxalenye yophando, oluyakuthatha intsuku ezintlanu ukuya kwisixhenxe zokuhanjelwan ngo Nompilo bakwa TB/HIV Care kwithuba lwenyanga ezine. Olutyelelo luyakufana nokuhambela kwamakhaya rhoqo okwenziwa ngooNompilo ukuxhasa abantu abaphila nentsholongwane kagawulayo ezindlwini zabo. Kolutyelelo rhoqo, siyakuthi sikucele uthathe inxaxheba ngokusabela ngenkcukacha nakwindlela equlunqiweyo yokukunceda ukuthi umelane nentsholongwane kagawulayo nokuthi ulawule ngcono indlela otya ngayo amayeza akho.

Ngaba uthatho nxaxheba lwam koluphando luyakugcinwa ngasese?

Abaphandi bazama ukukhusela ubuni kunye nobume begalelo lakho. Ukuqinisekisa ukungaziwa kwakho impendulo oyakuzinika koluphando zakuhlala ziyimfihlo kwaye asisayi kwabelana namntu okanye qela elingabandakanyekanga koluphando. Unelungelo lokuthi ufikelele kwinkcukacha zesisifundo okanye ucele utshintsho kwiinkcukhanca. Ukuqinekisa ukufihlwa kwakho iziphumo zaphando ziyakubhengezwa ngokungachazwanga kwintlanganiso yendibano yezobugqi.

Ngaba zithini iingozi koluphando?

Zonke iintsebenziswano zabantu kwakunye nokuthetha nabanye abantu ngesiqu sakho kuhlala kuneengozi. Kodwa ke sizakwenza ngako konke esinakho ukunciphisa ingozi ezinjalo kwaye sikhawulezise ukunceda ukuba uthe wafumana ukungaphatheki kakuhle, ngokwasengqondweni okanye nangeyiphi enye

Private Bag X17, Bellville, 7535
South Africa
Tel: +27 (0) 21 959 2809/2132
Fax: +27 (0) 21 9592872
Website: http://www.uwc.ac.za/faculties/chs/soph

indlela ngethuba lothatho nxaxheba lwakho koluphando. Apho kukho imfuneko, uyakuthi uthunyelwe kwinkcubabuchopho ukuze iqhubekeke ngoncedo lwakho. Ukwanalo nelungelo lokwala ukuphendula imibuzo ethile ukuba ezo nkcukacha zibuthathaka.

Ngaba zithini iingozi koluphando?

Zonke iintsebenziswano zabantu kwakunye nokuthetha nabanye abantu ngesiqu sakho kuhlala kuneengozi. Kodwa ke sizakwenza ngako konke esinakho ukunciphisa ingozi ezinjalo kwaye sikhawulezise ukunceda ukuba uthe wafumana ukungaphatheki kakuhle, ngokwasengqondweni okanye nangeyiphi enye indlela ngethuba lothatho nxaxheba lwakho koluphando. Apho kukho imfuneko, uyakuthi uthunyelwe kwinkcubabuchopho ukuze iqhubekeke ngoncedo lwakho. Ukwanalo nelungelo lokwala ukuphendula imibuzo ethile ukuba ezo nkcukacha zibuthathaka.

Ziziphi iinzuzo zoluphando?

Akukho zibonelelo ezihambelana nokuthatha inxaxheba kulolu cwaningo. Inkcukacha esiyakuthi sizifumane koluphando ziyakusenza sikwazi ukubonelela ngolwazi ukuphucula ukuphunyezwa kwenkxaso yokuncedisa unyango.

Ngaba ndinyanzelekile ukuba koluphando kwaye ndingathatha isigqibo sokurhoxa nanini na?
Ukuthatha inxaxheba kwakho koluphando kungokuzithandela ngokupheleleyo. Ukuba uthe wathatha isigqibo sokuthatha inxaxheba koluphando, ungaphinda uthathe isigqibo sokurhoxa nanini na, awusayi kohlwaywa okanye uphoswe yimivuso obunokuthi uyifumane.
Ukuba ngaba ndinemibuzo?
Oluphando luqhutywa ngu Njingalwazi Lucia Knight, School of Public Health kwi University ye Ntshona Koloni. Ukuba unemibuzo ngoluphando, nceda unxulumane no Njingalwazi Lucia Knight we School of Public Health kulo mnxeba: 021-5952243 and Email: lknight@uwc.ac.za

Ukuba ungaba nemibuzo malunga noluphando okanye amalungelo akho njengomthathi nxaxheba okanye unqwenela ukuxela ngengxaki othe wahlangabezana nazo ngokoluphando, nceda uxhulumane no:

Prof Uta Lehmann
School of Public Health
Head of Department
University of the Western Cape
Private Bag X17
Bellville 7535
soph-comm@uwc.ac.za

Prof Anthea Rhoda
Dean of the Faculty of Community and Health Sciences

FACULTY OF COMMUNITY AND HEALTH SCIENCES

Private Bag X17, Bellville, 7535
South Africa
Tel: +27 (0) 21 959 2809/2132
Fax: +27 (0) 21 9592872
Website:
http://www.uwc.ac.za/faculties/chs/soph

School of Public Health

Universiteit Antwerpen

University of the Western Cape
Private Bag X17
Bellville 7535
chs-deansoffice@uwc.ac.za

This research has been approved by the University of the Western Cape's Biomedical Research Ethics Committee.

Biomedical Research Ethics Committee
University of the Western Cape
Private Bag X17
Bellville
7535
Tel: 021 959 4111
e-mail: research-ethics@uwc.ac.za

FACULTY OF COMMUNITY AND HEALTH SCIENCES

School of Public Health

Private Bag X17, Bellville, 7535
South Africa
Tel: +27 (0) 21 959 2809/2132
Fax: +27 (0) 21 9592872
Website: http://www.uwc.ac.za/faculties/chs/soph

Universiteit Antwerpen

IFOMU YESIVUMELWANO

Isihloko seprojekti yophando: **Amakhaya akukhathalelo lwentsholngane kagawulayo:**

Olu phando ndilucaciselwe ngolwimi endiluqondayo. Imibuzo yam ngoluphando iye yaphenduleka. Ndiyaqonda ukuba luthetha ntoni uthatho nxaxheba lwam kwaye ndiiyavuma ukuthabatha inxhaxeba ngaphandle kwesinyanzelo. Ndiyaqonda ukuba ubumi bam abusayi kuvezwa nakubani na. Ndiyaqonda uukuba ndingabuya umva nangaliphi ixesha kuthatho nxaxheba ngaphandle kokunika izizathu noloyiko lweziphumo ezigwenxa nokuphoswa yinzuzo.

Igama lomthathi nxaxheba…………………………………………………………………………………………..

Utyikityo lomthathi nxaxheba…………………………………………………………………………………

Umhla………
