# Supplementary material for: Sinako, a study on HIV competent households in South Africa: a cluster-randomised controlled trial protocol
Source: Trials. 2020 Feb 10;21:154. doi: 10.1186/s13063-020-4082-0 (PMC7011384; doi:10.1186/s13063-020-4082-0)
Supplement: Supplementary file 2 — Additional file 2. Consent forms. [file 13063_2020_4082_MOESM2_ESM.zip › BASELINE INTERVENTION INT.pdf]

FACULTY OF COMMUNITY AND HEALTH SCIENCES

School of Public Health

Private Bag X17, Bellville, 7535
South Africa
Tel: +27 (0) 21 959 2809/2132
Fax: +27 (0) 21 9592872
Website: http://www.uwc.ac.za/faculties/chs/soph

Universiteit Antwerpen

INFORMATION LEAFLET: BASELINE INTERVENTION INTERVIEWS

Project title: Households in HIV care: an intervention to capitalize on the intermediate role of the household in community support for HIV care

Dear Sir/Madam,

What is this study about?

We, researchers from the University of the Western Cape, in collaboration with researchers of the University of Antwerp (Belgium) are doing research on household support for persons living with HIV. We are inviting you to participate in this research project because you have tested positive for HIV and have started treatment. The purpose of this research project is to understand your experience of living with HIV and being on treatment.

What will I be asked to do if I agree to participate?

We are inviting you to participate in this research study because your contributions will help us to understand how people living with HIV could be supported and possible in the future help others who find themselves in similar circumstances. To start with, we would like to conduct an interview with you where we will ask you to answer some questions about you, your household, your HIV test, disclosure of your status to your family, antiretroviral treatment, family and community support and other important issues about living with HIV. This interview is expected to take about an hour and a half of your time and we will use a cell phone to record your responses.

Once this interview is completed we would like to request that you participate in our study, which will involve about five to seven home visits with community health workers from TB/HIV Care that will take place over a period of about four months. These visits will be similar to the regular home visits that are carried out by community health workers to support people living with HIV in their houses. During these regular visits, we would ask you to take part in more information sharing and participatory exercises designed to help you to cope with your HIV and manage better adherence to your medication.

At the end of about six months, you will be asked to respond to another round of questions

Would my participation in this study be kept confidential?

The researchers undertake to protect your identity and the nature of your contribution. To ensure your anonymity, the answers you provide in this study will remain confidential and will not be viewed by or shared with any person or party not involved in this study. You are allowed to access the data and can ask for adaptations. To ensure your confidentiality, results will be anonymously published and presented at a meeting and scientific congress.

What are the risks of this research?

Private Bag X17, Bellville, 7535
South Africa
Tel: +27 (0) 21 959 2809/2132
Fax: +27 (0) 21 9592872
Website: http://www.uwc.ac.za/faculties/chs/soph

All human interactions and talking about self or others carry some amount of risks. We will nevertheless minimise such risks and act promptly to assist you if you experience any discomfort, psychological or otherwise during the process of your participation in this study. Where necessary, an appropriate referral will be made to a suitable professional for further assistance or intervention.

You are however free to decline to answer any specific question if you feel the information is too sensitive or personal.

What are the benefits of this research?
There are no direct benefits associated with your participation in this research. The data that we obtain from the study will enable us to provide information to improve the implementation of treatment adherence support.

Do I have to be in this research and may I stop participating at any time?
Your participation in this research is completely voluntary. You may choose not to take part at all. If you decide to participate in this research, you may stop participating at any time. If you decide not to participate in this study or if you stop participating at any time, you will not be penalized or lose any benefits to which you otherwise qualify.

What if I have questions?
This research is being conducted by Prof. Lucia Knight, School of Public Health at the University of the Western Cape. If you have any questions about the research study itself, please contact Prof. Lucia Knight. School of Public at Tel: 021-5952243 and Email: lknight@uwc.ac.za

Should you have any questions regarding this study and your rights as a research participant or if you wish to report any problems you have experienced related to the study, please contact:

Prof Uta Lehmann
School of Public Health
Head of Department
University of the Western Cape
Private Bag X17
Bellville 7535
soph-comm@uwc.ac.za

Prof Anthea Rhoda
Dean of the Faculty of Community and Health Sciences
University of the Western Cape
Private Bag X17
Bellville 7535

FACULTY OF COMMUNITY AND HEALTH SCIENCES

School of Public Health

Private Bag X17, Bellville, 7535
South Africa
Tel: +27 (0) 21 959 2809/2132
Fax: +27 (0) 21 9592872
Website:
http://www.uwc.ac.za/faculties/chs/soph

Universiteit Antwerpen

chs-deansoffice@uwc.ac.za

This research has been approved by the University of the Western Cape's Biomedical Research Ethics Committee.

Biomedical Research Ethics Committee
University of the Western Cape
Private Bag X17
Bellville
7535
Tel: 021 959 4111
e-mail: research-ethics@uwc.ac.za

FACULTY OF COMMUNITY AND HEALTH SCIENCES

School of Public Health

Private Bag X17, Bellville, 7535
South Africa
Tel: +27 (0) 21 959 2809/2132
Fax: +27 (0) 21 9592872
Website: http://www.uwc.ac.za/faculties/chs/soph

Universiteit Antwerpen

CONSENT FORM

Title of Research Project: ***Households in HIV care: an intervention to capitalize on the intermediate role of the household in community support for HIV care***

The study has been described to me in language that I understand. My questions about the study have been answered. I understand what my participation will involve and I agree to participate of my own choice and free will. I understand that my identity will not be disclosed to anyone. I understand that I may withdraw from the study at any time without giving a reason and without fear of negative consequences or loss of benefits.

Participant's name……………………………………………………………………………………………

Participant's signature…………………………………………………………………………………..

Date…….
