# Supplementary material for: Sinako, a study on HIV competent households in South Africa: a cluster-randomised controlled trial protocol
Source: Trials. 2020 Feb 10;21:154. doi: 10.1186/s13063-020-4082-0 (PMC7011384; doi:10.1186/s13063-020-4082-0)
Supplement: Supplementary file 2 — Additional file 2. Consent forms. [file 13063_2020_4082_MOESM2_ESM.zip › HOUSEHOLD MEMBER BASELINE INT.pdf]

FACULTY OF COMMUNITY AND HEALTH SCIENCES

School of Public Health

Private Bag X17, Bellville, 7535
South Africa
Tel: +27 (0) 21 959 2809/2132
Fax: +27 (0) 21 9592872
Website: http://www.uwc.ac.za/faculties/chs/soph

Universiteit Antwerpen

INFORMATION LEAFLET: HOUSEHOLD MEMBER BASELINE INTERVIEW

Project title: **An intervention to capitalize on the intermediate role of the household in community support for chronic diseases**

Dear Sir/Madam,

What is this study about?

We, researchers from the University of the Western Cape, in collaboration with researchers of the University of Antwerp (Belgium) are doing research on the experiences of households and people like you in your community and particularly how chronic illness affects them. We are inviting you to participate in this research project because we want to explore your experiences and perspectives around the care and support of someone living with a chronic disease in your houdehold.

What will I be asked to do if I agree to participate?

We are inviting you to participate in this research study because of how valuable your contributions will be to help us understand how the members of a household could support others having chronic diseases in the households and community. In this study, we will ask you some questions about you, your household, family and community support and other important issues about illness in your household. This interview is expected to take about an hour and a half of your time and we will use a cell phone to record your responses as we go through the questions. We might request to ask you questions on the same subject some at some other time in the future. We hope that you would be willing to help us.

Would my participation in this study be kept confidential?

The researchers undertake to protect your identity and the nature of your contribution. To ensure your anonymity, the answers you provide in this study will remain confidential and will not be viewed by or shared with any person or party not involved in this study. You are allowed to access the data and can ask for adaptations. To ensure your confidentiality, results will be anonymously published and presented at a meeting and scientific congress.

What are the risks of this research?

All human interactions and talking about self or others carry some amount of risks. We will nevertheless minimise such risks and act promptly to assist you if you experience any discomfort, psychological or otherwise during the process of your participation in this study. Where necessary, an appropriate referral will be made to a suitable professional for further assistance or intervention. You are however free to decline to answer any specific question if you feel the information is too sensitive or personal.

What are the benefits of this research?

There are no direct benefits associated with your participation in this research. The data that we obtain from the study will enable us to provide information to improve the implementation of treatment adherence support.

Private Bag X17, Bellville, 7535
South Africa
Tel: +27 (0) 21 959 2809/2132
Fax: +27 (0) 21 9592872
Website: http://www.uwc.ac.za/faculties/chs/soph

Do I have to be in this research and may I stop participating at any time?

Your participation in this research is completely voluntary. You may choose not to take part at all. If you decide to participate in this research, you may stop participating at any time. If you decide not to participate in this study or if you stop participating at any time, you will not be penalized or lose any benefits to which you otherwise qualify.

What if I have questions?

This research is being conducted by Prof. Lucia Knight, School of Public Health at the University of the Western Cape. If you have any questions about the research study itself, please contact Prof. Lucia Knight. School of Public at Tel: 021-5952243 and Email: lknight@uwc.ac.za

Should you have any questions regarding this study and your rights as a research participant or if you wish to report any problems you have experienced related to the study, please contact:

Prof Uta Lehmann
School of Public Health
Head of Department
University of the Western Cape
Private Bag X17
Bellville 7535
soph-comm@uwc.ac.za

Prof Anthea Rhoda
Dean of the Faculty of Community and Health Sciences
University of the Western Cape
Private Bag X17
Bellville 7535
chs-deansoffice@uwc.ac.za

This research has been approved by the University of the Western Cape's Biomedical Research Ethics Committee.

Biomedical Research Ethics Committee
University of the Western Cape
Private Bag X17
Bellville
7535
Tel: 021 959 4111
e-mail: research-ethics@uwc.ac.za

FACULTY OF COMMUNITY AND HEALTH SCIENCES

School of Public Health

Private Bag X17, Bellville, 7535
South Africa
Tel: +27 (0) 21 959 2809/2132
Fax: +27 (0) 21 9592872
Website: http://www.uwc.ac.za/faculties/chs/soph

Universiteit Antwerpen

CONSENT FORM

Title of Research Project: ***An intervention to capitalize on the intermediate role of the household in community support for chronic diseases***

The study has been described to me in language that I understand. My questions about the study have been answered. I understand what my participation will involve and I agree to participate of my own choice and free will. I understand that my identity will not be disclosed to anyone. I understand that I may withdraw from the study at any time without giving a reason and without fear of negative consequences or loss of benefits.

Participant's name………………………………………………………………………………………

Participant's signature……………………………………………………………………………….

Date……….
