# Supplementary material for: Sinako, a study on HIV competent households in South Africa: a cluster-randomised controlled trial protocol
Source: Trials. 2020 Feb 10;21:154. doi: 10.1186/s13063-020-4082-0 (PMC7011384; doi:10.1186/s13063-020-4082-0)
Supplement: Supplementary file 2 — Additional file 2. Consent forms. [file 13063_2020_4082_MOESM2_ESM.zip › HOUSEHOLD MEMBER BASELINE INT_Afrikaans.pdf]

FACULTY OF COMMUNITY AND HEALTH SCIENCES

Private Bag X17, Bellville, 7535
South Africa
Tel: +27 (0) 21 959 2809/2132
Fax: +27 (0) 21 9592872
Website: http://www.uwc.ac.za/faculties/chs/soph

School of Public Health

Universiteit Antwerpen

INFORMASIE BLAD: BASISLYN BEHEER ONDERHOUDE

Projek titel: **Huishoudings in MIV-sorg: 'n intervensie om te kapitaliseer op die intermediêre rol van die huishouding in gemeenskapsondersteuning vir MIV-sorg**

Geagte Meneer/Mevrou,

Waaroor is hierdie studie?

Ons, as navorsers van die Universiteit van Wes-Kaapland, in samewerking met navorsers van die Universiteit van Antwerpen (België), doen navorsing oor die ervarings van huishoudings en mense soos jouself in jou gemeenskap en veral hoe chroniese siektes hulle raak. Ons nooi u uit om deel te neem aan hierdie navorsingsprojek, want ons wil u ervaring en perspektief oor die versorging en ondersteuning van iemand met 'n chroniese siekte in ‘n hiuishouding ondersoek.

Wat sal ek gevra word om te doen as ek instem om deel te neem?

Ons nooi u uit om deel te neem aan hierdie navorsingsstudie weens hoe waardevol u bydrae sal wees om ons te help verstaan hoe die lede van 'n huishouding ander met chroniese siektes in die huishouding, en gemeenskap, kan ondersteun. In hierdie studie sal ons u vrae stel oor u, u huishouding, familie- en gemeenskapsondersteuning en ander belangrike kwessies rakende siekte in u huishouding. Hierdie onderhoud sal na verwagting sowat 'n uur en ‘n half van u tyd neem en ons sal 'n selfoon gebruik om u antwoorde op te neem terwyl ons deur die vrae gaan. Ons mag dalk oor ‘n paar maande weer vrae vra oor hierdie selfde onderwerp. Ons hoop dat u bereid sal wees om ons hiermee te help.

Sal my deelname aan hierdie studie vertroulik gehou word?

Die navorsers onderneem om u identiteit en die aard van u bydrae te beskerm. Om u anonimiteit te verseker, sal die antwoorde wat u in hierdie studie verskaf, vertroulik bly en sal nie gesien of gedeel word met enige persoon of party wat nie by hierdie studie betrokke is nie. U het toegang tot die data en kan vir aanpassings vra. Om u vertroulikheid te verseker, sal die uitslae anoniem gepubliseer word en op 'n vergadering en wetenskaplike kongres aangebied word.

Wat is die risiko's van hierdie navorsing?

Alle menslike interaksies en praat oor self of ander dra 'n aantal van risiko's. Ons sal egter sulke risiko's verminder en dadelik optree om u te help as u enige ongemak, sielkundige of andersins ervaar tydens die proses van u deelname aan hierdie studie. Indien nodig sal 'n gepaste verwysing na 'n geskikte professionele persoon en/of instansie gedoen word vir verdere hulp of ingryping. U kan egter weier om enige spesifieke vraag te beantwoord as u voel dat die inligting te sensitief of persoonlik is.

Private Bag X17, Bellville, 7535
South Africa
Tel: +27 (0) 21 959 2809/2132
Fax: +27 (0) 21 9592872
Website:
http://www.uwc.ac.za/faculties/chs/soph

Universiteit Antwerpen

Wat is die voordele van hierdie navorsing?

Daar is geen direkte voordele verbonde aan jou deelname aan hierdie navorsing nie. Die data wat ons van die studie verkry, sal ons in staat stel om inligting te verskaf om die implementering van behandeling-bystandsondersteuning te verbeter.

Moet ek in hierdie navorsing wees en mag ek op enige stadium ophou deelneem?

U deelname aan hierdie navorsing is heeltemal vrywillig. U mag kies om glad nie deel te neem nie. As u besluit om aan hierdie navorsing deel te neem, kan u enige tyd u deelname stop. As u besluit om nie aan hierdie studie deel te neem nie, of as u op enige stadium ophou deelneem, sal u nie gepenaliseer word, of enige voordele wat u andersins voor kwalifiseer, verloor nie.

Wat as ek vrae het?

Hierdie navorsing word deur prof. Lucia Knight, Skool vir Openbare Gesondheid aan die Universiteit van Wes-Kaapland, uitgevoer. As u vrae het oor die navorsingstudie self, kontak asseblief prof. Lucia Knight. Skool van Openbare by Tel: 021-5952243 en E-pos: lknight@uwc.ac.za

Indien u enige vrae rakende hierdie studie en u regte as navorsingsdeelnemer het of as u enige probleme rakende die studie aangemeld wil hê, kontak asseblief:

Prof Uta lehmann
Skool vir Openbare Gesondheid
Hoof van die departement
Universiteit van die Wes-Kaap
Privaatsak X17
Bellville 7535
soph-comm@uwc.ac.za

Prof Anthea Rhoda
Dekaan van die Fakulteit Gemeenskaps- en Gesondheidswetenskappe
Universiteit van die Wes-Kaap
Privaatsak X17
Bellville 7535
chs-deansoffice@uwc.ac.za

This research has been approved by the University of the Western Cape's Biomedical Research Ethics Committee.
Biomedical Research Ethics Committee
University of the Western Cape
Private Bag X17
Bellville
7535
Tel: 021 959 4111

FACULTY OF COMMUNITY AND HEALTH SCIENCES

School of Public Health

Private Bag X17, Bellville, 7535
South Africa
Tel: +27 (0) 21 959 2809/2132
Fax: +27 (0) 21 9592872
Website:
http://www.uwc.ac.za/faculties/chs/soph

Universiteit Antwerpen

VRYWARINGS VORM

Titel van Navorsingsprojek: ***Huishoudings in MIV-sorg: 'n intervensie om te kapitaliseer op die intermediêre rol van die huishouding in gemeenskapsondersteuning vir MIV-sorg***

Die studie is aan my beskryf in taal wat ek verstaan. My vrae oor die studie is beantwoord. Ek verstaan wat my deelname sal behels en ek stem in om deel te neem van my eie keuse en vrye wil. Ek verstaan dat my identiteit nie aan enigiemand bekend gemaak sal word nie. Ek verstaan dat ek enige tyd van die studie kan onttrek sonder 'n rede en sonder vrees vir negatiewe gevolge of verlies aan voordel.

Deelnemer se naam ....

Deelnemer se handtekening ....

Datum ....
